# Tegaserod Maleate Inhibits Breast Cancer Progression and Enhances the Sensitivity of Immunotherapy

**DOI:** 10.1155/2022/5320421

**Published:** 2022-02-03

**Authors:** Xiao Li, Liangliang Wu, Zhiying Zheng, Hanyuan Liu, Haiyang Li, Guoqiang Sun, Guangshun Sun, Ye Cheng, Hanjin Wang, Zhouxiao Li, Junfeng Shi, Weiwei Tang

**Affiliations:** ^1^Department of General Surgery, Nanjing First Hospital, Nanjing Medical University, Nanjing, Jiangsu, China; ^2^Department of Anesthesiology, The First Affiliated Hospital of Nanjing Medical University, Nanjing, Jiangsu, China; ^3^Department of General Surgery, Nanjing Drum Tower Hospital, The Affiliated Hospital of Nanjing University Medical School, Nanjing, Jiangsu, China; ^4^Division of Hand, Plastic and Aesthetic Surgery, University Hospital LMU, Munich, Germany; ^5^Department of Oncology, Nanjing First Hospital, Nanjing Medical University, Nanjing, Jiangsu, China; ^6^Hepatobiliary/Liver Transplantation Center, The First Affiliated Hospital of Nanjing Medical University, Key Laboratory of Living Donor Transplantation, Chinese Academy of Medical Sciences, Nanjing, Jiangsu, China

## Abstract

**Background:**

Breast cancer (BC) is the most commonly diagnosed cancer in women worldwide. The challenge in managing this heterogeneous malignancy is that BC is highly aggressive and is always associated with chemical resistance, radiation resistance, hormone therapy resistance, and targeted therapy resistance. Therefore, there is an urgent need to find effective drugs to treat BC.

**Methods:**

Based on the Selleck drug library approved by FDA, we screened 800 drugs for anti-BC cells and found that tegaserod maleate (TM), a 5-hydroxytryptamine 4-receptor (HTR4) partial agonist had the best anti-BC effect, which was further verified. The effects of different concentrations of TM on cell proliferation, invasion, and migration were evaluated in vitro using CCK8, plate cloning, transwell, and scratch assays. The UALCAN database, Kaplan–Meier Plotter database, Human Protein Atlas, and GEPIA2 were used to explore the correlation between HTR4 expression and BC patients' clinicopathological data as well as immune response. In vivo experiments demonstrated the effect of the TM and immunotherapy drug (anti-PD1/anti-TIGIT) combination on BC tumor growth in mice.

**Results:**

TM significantly inhibited the proliferation, invasion, and migration of BC cells, and the higher the concentration, the better the inhibition effect. HTR4 was significantly downregulated in BC tissues compared to paracancerous tissues. The downregulation of HTR4 was correlated with clinicopathological data and positively correlated with BC prognosis. Interestingly, the GEPIA2 database suggested that there was a strong positive correlation between the expression of HTR4 and effector T cells, effector memory T cells, and exhausted T cells. In vitro experiments showed that TM, anti-PD1, and anti-TIGIT could all inhibit the growth and weight of BC tumors as compared with the control group. However, when anti-PD1 or anti-TIGIT was used simultaneously with TM, the inhibition of tumors significantly exceeded that in the control group. Moreover, the combination of anti-TIGIT and TM has the best inhibitory effect.

**Conclusion:**

TM inhibited the progression of breast cancer, and its combination with anti-TIGIT could effectively inhibit tumor growth and improve the sensitivity of immunotherapy in breast cancer.

## 1. Introduction

Breast cancer (BC) is the most commonly diagnosed cancer in women worldwide [1], and a complex disease with morphological and molecular heterogeneity, characterized by three morphological grades and more than four different molecular subtypes (at the level of gene expression) [2]. According to the consensus reached in 2015 and 2017 St. Gallen International Breast Cancer Expert Conference [3, 4], BC is clinically divided into four subtypes: triple negative, hormone receptor (HR) negative and human epidermal growth factor receptor 2 (HER2) positive, HR positive and HER2 positive, HR positive, and HER2 negative. Treatment options vary widely for different types of BC.

One hallmark of cancer including BC is the ability to evade the immune system through tumor-mediated immune escape [5]. Immune evasion can occur through a variety of mechanisms, including manipulation of key immune checkpoints that regulate the adaptive immune system, including cytotoxic lymphocyte antigen 4 (CTLA-4), programmed cell death protein 1 (PD-1), and programmed cell death 1 ligand 1 (PD-L1). The CTLA-4 inhibitor was the first immune checkpoint inhibitor (ICI) to demonstrate a benefit in melanoma; subsequently, response rates for PD-1/PD-L1 inhibitors varied from 10% to 27% for multiple cancers [6–8]. T cell immune receptor with Ig and ITIM domains (TIGIT) is a type I transmembrane protein expressing Ig-like variable extracellular domain (NKT) on activating and memory T cells, regulatory T cells, natural killer (NK), and natural killer T cells (NKT) [9]. Although the U.S. Food and Drug Administration (FDA) has approved multiple ICI for many cancers, the percentage of patients benefiting from monotherapy ICI is low. Therefore, the strategy of pursuing ICI combined with other drugs is of great significance for the treatment of cancers including BC.

Repurposing existing drugs is a time-saving way to develop drugs that are more effective and have fewer side effects. There is substantial evidence that FDA-approved nonantineoplastic drugs, such as antibiotics, anti-inflammatory drugs, lipid-lowering agents, and sulfonylureas, have been shown to have antitumor effects in a variety of cancers [10]. In this study, we screened 800 drugs against BC cells based on the Selleck drug library approved by the FDA. We were fortunate to find that tegaserod maleate (TM), a partial agonist of 5-hydroxytryptamine 4-receptor (HTR4), worked best on anti-BC cells, which was confirmed in subsequent studies.

## 2. Materials and Methods

### 2.1. Cell Culturing and Compounds

Selleck drug library approved by FDA including TM was purchased from Selleck Chemicals LLC (USA). After dissolving in dimethyl sulfoxide (DMSO, Gibco, USA) to a final concentration of 10 mM, TM was aliquoted and stored at −80°C. MDA-MB-231, MCF-7, and ZR75-1 cells received the culturing process using RPMI DMEM medium (Gibco, USA) involving 10% fetal bovine serum (FBS) (Gibco, USA) under 37°C inside one 5% CO_2_ chamber covering streptomycin (100 mg/mL) and penicillin (100 IU/mL). Half-maximal inhibitory concentration (IC50) values were computed from dose-response curves using Excel.

### 2.2. Cell Proliferation Experiments

In the Cell Counting Kit-8 (CCK-8) test, MDA-MB-231, MCF-7, and ZR75-1 cells were cultured with TM (2.5 *μ*M and 5 *μ*M) at 37°C. Next, after the medium was replaced, the CCK-8 solution (Biosharp, China) was introduced into each well and incubated for 2 h. The absorbance was measured at 450 nm at 0, 24, 48, 72, and 96 hours. During the clone formation experiment, cells were exposed to TM at different concentrations (2.5 *μ*M and 5 *μ*M) for 24 h and then inoculated in 6-well plates at a density of 1000 cells per well. After 10 days, the cells were imfixed based on the use of methanol and then stained with Gimsa (Wobixin Inc., China). Finally, the colony was imaged and counted.

### 2.3. Transwell Assay

MDA-MB-231, MCF-7, and ZR75-1 cells were inoculated with 200 *μ*l serum-free RPMI 1640 medium and TM (2.5 *μ*M and 5 *μ*M) at different concentrations. Transwell Cell (Corning, USA) underwent an intrusion testing process using a paving process with matrix glue mixture (BD Biosciences, USA) and did not use matrix glue mixture for migration testing. RPMI DMEM medium and 10% FBS were introduced into the bottom chamber to act as BC cell chemical attractants. When the 24-hour culture process was completed, the upper chamber was immobilized and then stained for 15 minutes with crystal violet (Kagan, China). For the visualization program, the cell line receives the photo and counting program in three fields.

### 2.4. Wound Healing Assay

MDA-MB-231, MCF-7, and ZR75-1 cells received different concentrations of TM (2.5 *μ*M and 5 *μ*M) when the seeding on 6-well culture plates was achieved. Using a standard 20 *μ*l pipette tip, the artificial linear wound was eliminated on the fused cell monolayer. Free-floating cells and debris isolated from the bottom of the well were slowly removed. Medium was introduced and the plate was incubated at 37°C. Scratch widths were recorded under an inverted microscope and photographed at 0, 24, and 48 hours.

### 2.5. HTR4 Expression Level and Clinicopathological Analysis as well as Immune Analysis

UALCAN was used here to compare HTR4 expression in BC patients of different stages, histological subtypes, lymph node metastasis, and so on. The Human Protein Atlas was utilized for obtaining HTR4 protein expression in BC tissues. The Kaplan–Meier Plotter was used to compare correlations between HTR4 expression and overall survival (OS), distant metastasis-free survival (DMFS), relapse-free survival (RFS), and post-progression survival (PPS). GEPIA2 was applied to analyze the correlation between HTR4 expression and immune cells.

### 2.6. Mice Model

The animal management committee of Nanjing Medical University approved the animal experiment, and all experiment procedures and animal care conformed to the institutional ethics directions for animal-related experiments. 1 × 10^6^ 4T1 cells were inoculated into the right groin of BALB/C mice. The groups were PBS, TM, anti-PD1 (BioXcell, BP0273), anti-TIGIT (BioXcell, BE0274), TM + anti-PD1, and TM + anti-TIGIT (*n* = 5 for the respective group). 10 mg/kg TM intraperitoneal injection was made for TM group every three days. 6.6 mg/kg intraperitoneal injection was made for anti-PD1/anti-TIGIT group on the eight day, and once per three days thereafter. Tumor growth was observed. After 20 days, the mice were killed and the tumor tissue was taken out for weighing and immunohistochemical analysis.

### 2.7. Immunohistochemical Staining

Tumor tissues from mice were embedded in paraffin blocks for immunohistochemical staining and analysis. Tissues sections were deparaffinized and hydrated. Sections were incubated with 3% H2O2 for 10 min and then incubated at 4°C with primary antibodies (CD4, CD8, Ki67, PD1, and PD-L1) overnight. A secondary antibody was added and incubated at 37°C for 15 min. Tissue sections were stained using diaminobenzidine and hematoxylin. Finally, sections were dehydrated and covered with glass slides. All tissue sections were photographed using a microscope camera and analyzed using TissueFAXS Viewer software program.

### 2.8. Statistics-Related Analyzing Process

The continuing information received the comparative analysis by performing one individual *t*-testing process of the two groups. A statistics-related analyzing process was performed and presented graphically in GraphPad Prism 8.0 (USA). A *P*value of 0.05 was considered to be statistically significant.

## 3. Result

### 3.1. A Screen of the Selleck Drug Library Approved by the FDA Identified TM as Having Anti-BC Activity

**Figure 1:**
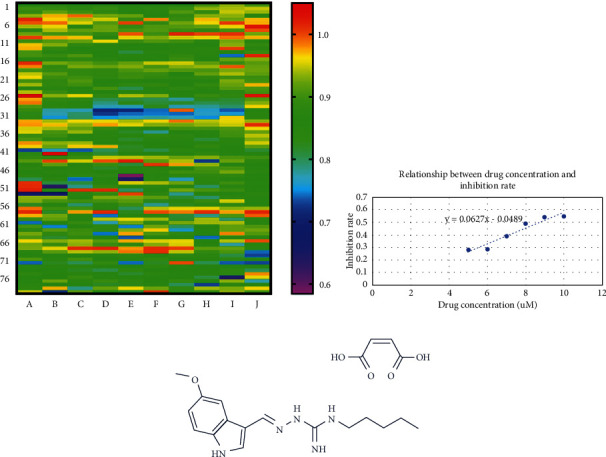
A screen of Selleck drug library approved by FDA identified TM having anti-BC activity. (a) A cell viability screening identified that TM had anti-BC activity among the compounds from the Selleck drug library approved by the FDA. (b) The linear graph of IC50 values in BC cells according to the CCK8 assay. (c) The chemical structure of TM.

To identify drugs with novel anti-BC activities using an unbiased approach, we screened the Selleck drug library approved by the FDA including 800 small molecules against the BC cell line. We found that TM had the best inhibitory effect on BC with a rate of 58.4% (Figure 1(a), Supplementary Table 1). Through the CCK8 assay, we found that the half-maximal inhibitory concentration (IC50) value in the MDA-MB-231 cell line was 8.75 *μ*M (Figure 1(b)). The chemical structure of TM is shown in Figure 1(c).

### 3.2. TM-Inhibited BC Progression

**Figure 2:**
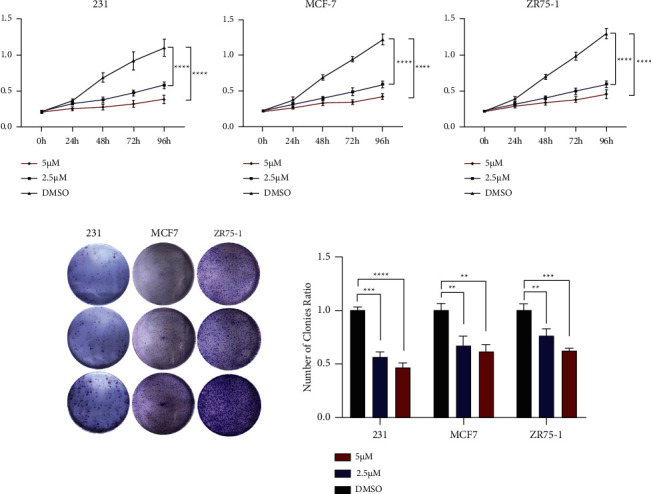
TM inhibited BC proliferation. (a) The CCK8 assay results at different concentrations of TM for BC cells. (b) Plate cloning experiment of different concentrations of TM for BC cells. ^∗∗^*P* < 0.01; ^∗∗∗^*P* < 0.001; ^∗∗∗∗^*P* < 0.0001.

**Figure 3:**
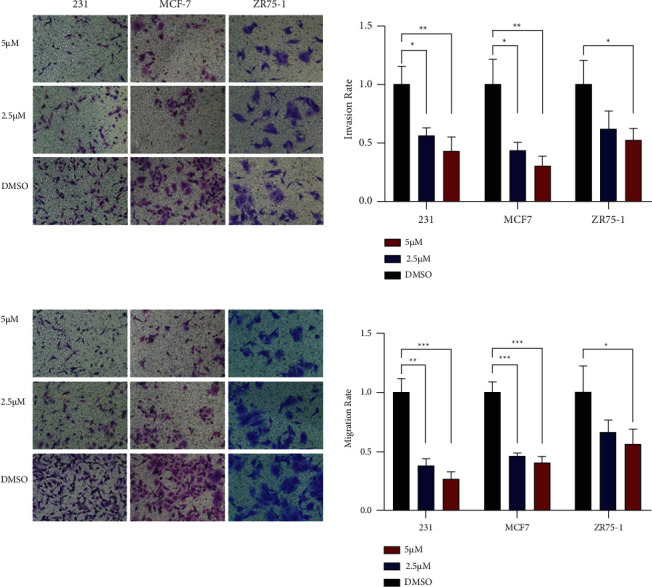
TM inhibited BC invasion and migration. (a) Different concentrations of TM could inhibit the invasion of BC cells via the transwell assay. (b) Different concentrations of TM could inhibit the migration of BC cells via the transwell assay. ^*∗*^*P* < 0.05; ^*∗∗*^*P* < 0.01; ^*∗∗∗*^*P* < 0.001.

**Figure 4:**
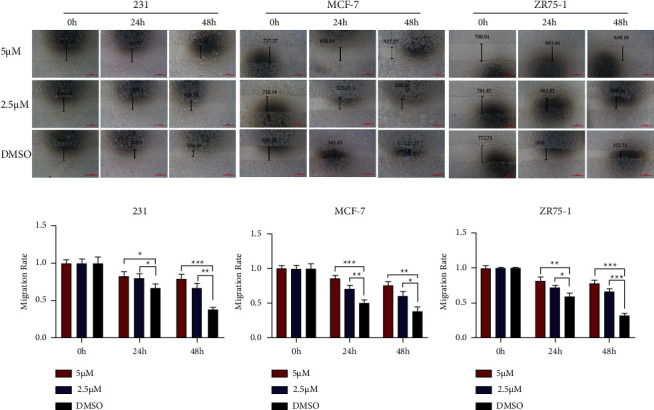
TM inhibited BC migration. (a) Different concentrations of TM could inhibit the migration of BC cells via the scratch assay. (b) The migration rate for each group. ^*∗*^*P* < 0.05; ^*∗∗*^*P* < 0.01; ^*∗∗∗*^*P* < 0.001.

We added 2.5 µM and 5 µM of TM, respectively, and evaluated the effect of TM on BC cells with DMSO dissolution as the control. The results of the CCK8 and plate cloning assay showed that TM significantly inhibited the proliferation of BC cells (MDA-MB-231, MCF-7, and ZR75-1) compared with the control group, and the higher the concentration of drugs added, the more obvious the inhibition effect (Figures 2(a) and 2(b)). The Transwell assay showed that compared with the control group, TM inhibited the migration and invasion rate of BC cells, and the higher the concentration of drugs added, the more obvious the inhibition effect (Figures 3(a) and 3(b)). The wound healing assay revealed that the scratch closure rate was significantly lower than that of the control group after adding TM (Figures 4(a) and 4(b)). These results all demonstrated that TM significantly inhibited the ability of three different types of BC cells to proliferate, invade, and migrate.

### 3.3. Clinical Role of HTR4 in BC Based on Database Analysis

**Figure 5:**
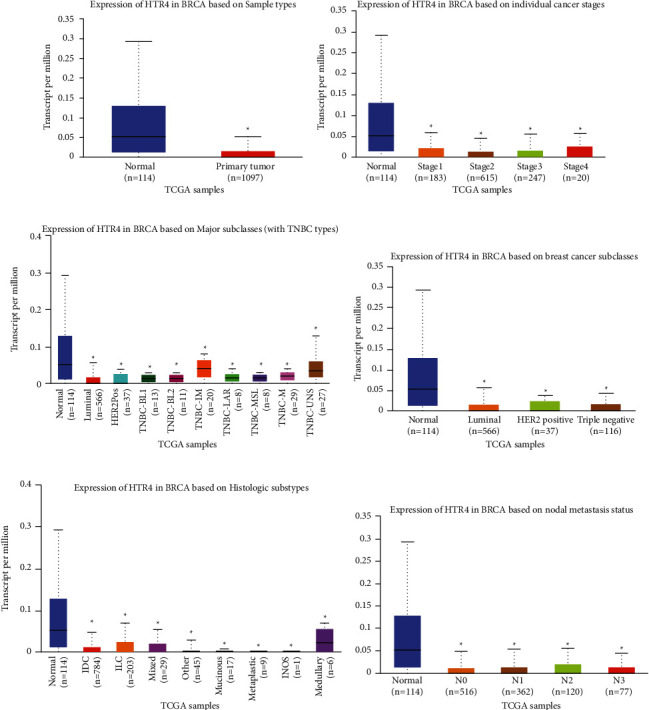
The clinical role of HTR4 in BC. (a–f)The correlation between HTR4 mRNA expression and BC sample types, tumor stage, subtypes, subclasses, histological subtypes, and lymph node metastatic status. ^*∗*^*P* < 0.05.

**Figure 6:**
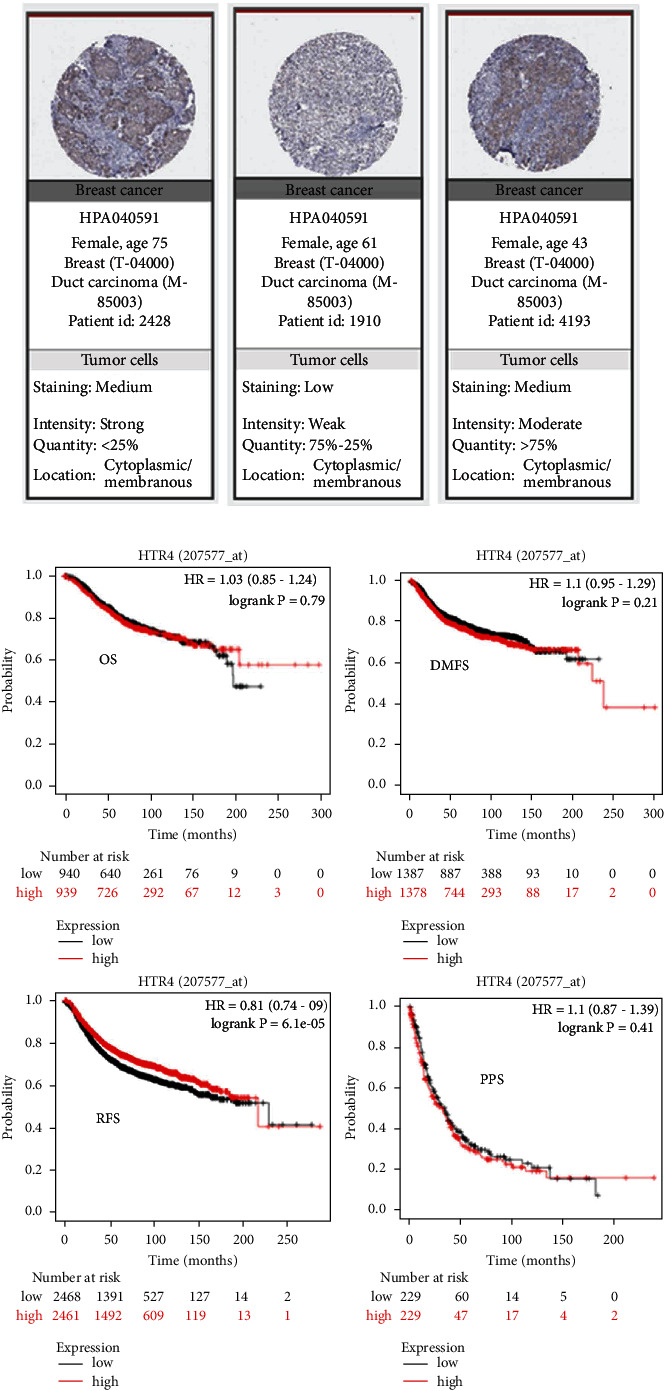
Expression and prognostic analysis of the HTR4 protein in BC. (a) The protein expression level of HTR4 in BC tissues. (b) The relationship between HTR4 expression and BC patients' OS, RFS, DMFS, and PPS (OS, overall survival; RFS, relapse-free survival; DMFS, distant metastasis-free survival; PPS, postprogression survival).

The target of TM excitation is HTR4. Therefore, we investigated the expression and function of HTR4 in BC tissues. The TCGA portal showed that the expression of HTR4 in tumor tissues was obviously lower than that in normal tissues (Figure 5(a)). Subgroup analysis based on BC subtypes, histological subtypes, stages, and lymph node metastasis showed that the expression of HTR4 decreased in different types of BC tissues compared with normal tissues, and the higher the cancer stage and lymph node metastasis, the lower the expression of HTR4 (Figures 5(b)–5(f)). Analysis of the Human Protein Atlas data indicated that HTR4 protein expression was moderate or low in BC patients (Figure 6(a)). The prognostic potential of HTR4 in BC was further examined using the Kaplan–Meier Plotter. The results showed that BC patients with low HTR4 expression were not associated with OS, DMFS, and PPS, but were significantly correlated with RFS (Figure 6(b)). These results suggested that HTR4 might play an important role in BC tissues.

### 3.4. HTR4 Expression Was Correlated with Immune Factors in BC

**Figure 7:**
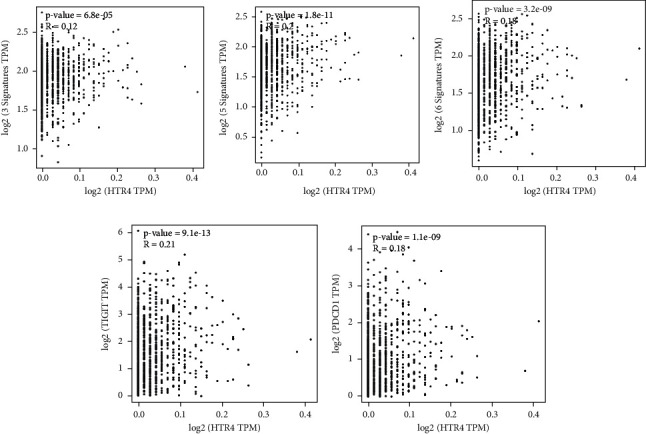
HTR4 expression was correlated with immune factors in BC. (a–c) Dot chart showing the correlation of HTR4 with effector T cells, effector memory T cells, and exhausted T cells in BC. (d, e) Dot chart showing the correlation of HTR4 with TIGIT and PD1 expression in BC.

Existing studies have confirmed that the immune system is closely related to the occurrence and development of tumors. Therefore, we investigated the relationship between the expression of HTR4 and immune factors. As shown in Figures 7(a)–7(c), there was a strong positive correlation between the expression of HTR4 and effector T cells, effector memory T cells, and exhausted T cells. More interestingly, HTR4 expression was significantly positively correlated with PD1 and TIGIT expression, well-known targets of CD8^+^ T cell exhaustion (Figures 7(d) and 7(e)). Therefore, we will further study the effect of TM on the immunotherapy of BC.

### 3.5. TM Enhances the Sensitivity of Immunotherapy in BC

**Figure 8:**
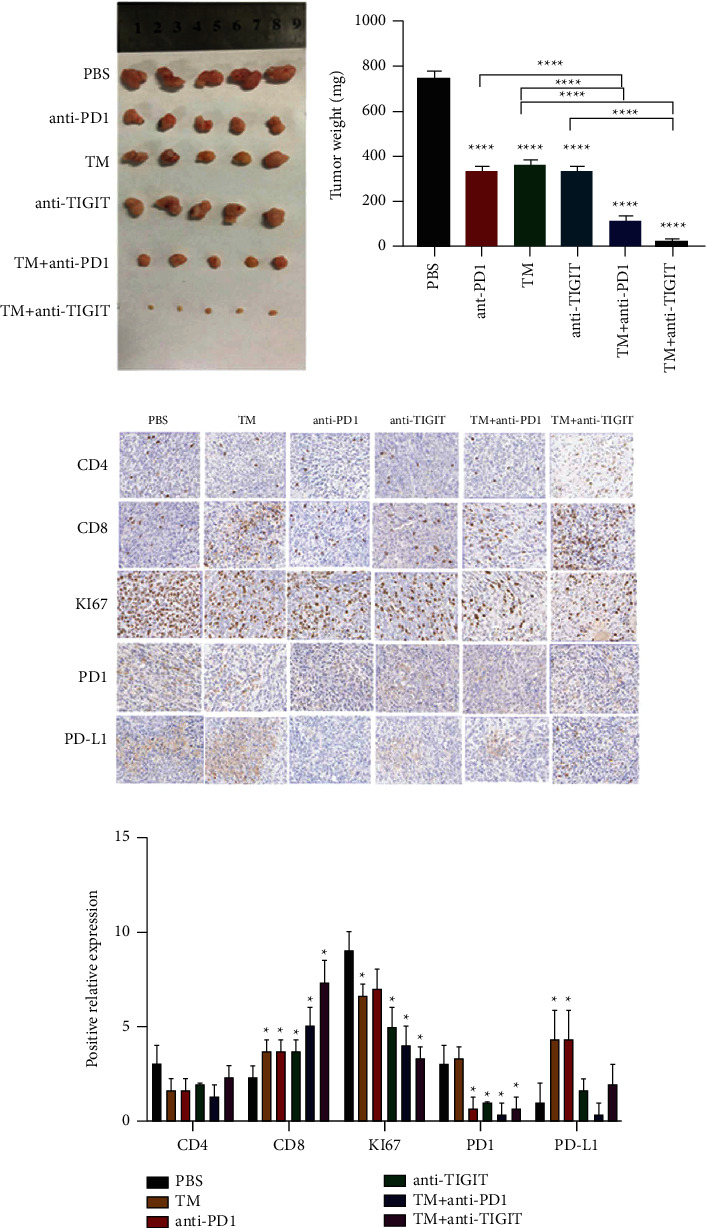
TM enhanced the sensitivity of immunotherapy in BC. (a) Picture display of the respective group of tumors. (b) Analysis of the weight of tumors in the respective group. (c) The tumors in each group were confirmed by HE staining. (d) HE staining analysis of each group. ^*∗*^*P* < 0.05; ^*∗∗*^*P* < 0.01; ^*∗∗∗*^*P* < 0.0001.

To examine the correlation between TM and the growth of BC in vivo, we injected 4T1 cells into the right groin of 30 BALB/C mice, respectively, and then carried out an anti-PD1/anti-TIGIT injection to assess their antitumor capacity. According to the results, TM, anti-PD1, and anti-TIGIT could all inhibit the growth and weight of tumors, as compared with the control group (Figures 8(a) and 8(b)). When anti-PD1 or anti-TIGIT were used simultaneously, the inhibition of tumor significantly exceeded that in the control group. Moreover, the combination of anti-TIGIT and TM had the best inhibitory effect (Figures 8(a) and 8(b)). Given the immunohistochemical results, the expression of CD4 in each group showed no significant difference (Figures 8(c) and 8(d)). Compared with the PBS group, the TM group significantly promoted the expression of CD8 and PD-L1, suggesting that the addition of TM activated the immune function of BC (Figures 8(c) and 8(d)). When TM was combined with anti-PD1/anti-TIGIT, the expression of CD8 was significantly increased, and the expression of KI67 and PD1 was significantly decreased (Figures 8(c) and 8(d)). Accordingly, this study revealed that TM was capable of reducing BC growth and increasing the efficiency of anti-PD1 or anti-TIGIT treatment in BC.

## 4. Discussion

TM is used for the treatment of constipation-type irritable bowel syndrome as a HTR4 partial agonist [11]. Studies have shown that 5-hydroxytryptamine plays a mitogenic role in colon cancer cells, and HTR4 is significantly expressed in both colon cancer tissue and cells [12]. Wu et al. reported that TM inhibited esophageal squamous cell carcinoma proliferation by suppressing the peroxisome pathway [13]. Zhang et al. found that TM could cause G1 cell cycle arrest, induce cell apoptosis, and inhibit the growth of a variety of cancer cells [14]. Liu et al. used a screen of 770 pharmacologically active and/or FDA-approved drugs and identified TM as a novel anticancer compound that can induce apoptosis of mouse and human malignant melanoma cell lines. The antiapoptotic induction effect of TM was unrelated to the serotonin signal and was attributed to PI3K/Akt/mTOR signal inhibition. TM reduced tumor growth and metastasis and increased survival in a model of in vivo homologous immune activity [15]. In our study, we screened the Selleck drug library approved by the FDA including 800 small molecules and found TM had the best anti-BC effect, which was further verified. TM significantly inhibited the proliferation, invasion, and migration of BC cells, and the higher the concentration, the better the inhibition effect. A search of the literature showed that this is the first report in the world that TM could be used against BC.

TIGIT has been reported to be coexpressed with PD-1 on tumor-antigen specific CD8^+^ T cells and CD8^+^ tumor-infiltrating lymphocytes (TIL) in human cancers [16]. In addition, coexpression of TIGIT and other inhibitory receptors on exhausted CD8^+^ T cell subsets in tumors, such as molecule 3 in the T cell immunoglobulin and mucin domain (TIM-3) and lymphocyte activation gene 3 (LAG-3), was observed [17]. Although multiple sources of evidence support the critical role of TIGIT in limiting tumor-specific adaptation and innate immunity, the role of TIGIT and its association with the tumor-immune microenvironment in BC remains largely unknown. Stamm Hauke et al. showed blocking TIGIT or PVR resulted in enhanced immune cell-mediated lysis of BC cell lines (SKBR-3, MDA-MB-231, MDA-MB-468, and BT549) and additionally increased the cytotoxic effects of a bispecific T cell engager BiTE® antibody construct targeting EGFR [18]. Xu Feng et al. reported that blockade of TIGIT or CD112 R, separately or together, enhanced the trastuzumab-triggered antitumor response by human NK cells. PVR-like receptors regulate NK cell functions and could be targeted for improving trastuzumab therapy for breast cancer [19]. In our study, in vitro experiments showed that TM, anti-PD1, and anti-TIGIT could all inhibit the growth and weight of BC tumors as compared with the control group. However, when anti-PD1 or anti-TIGIT was used simultaneously with TM, the inhibition of tumors significantly exceeded that in the control group. Moreover, the combination of anti-TIGIT and TM showed the best inhibitory effect. This conclusion adds a new bright spot for the application of TIGIT in cancer.

There are some shortcomings in our research. First, we did not coculture BC cells with immune cells to prove our conclusion. Secondly, we did not explore the mechanism by which TM activated the immune activity of BC. Thirdly, we do not use more advanced models such as the patient-derived xenografts (PDX) model to verify the validity of this conclusion in human samples.

## 5. Conclusion

Tegaserod maleate inhibits the progression of breast cancer, and its combination with anti-TIGIT can effectively inhibit tumor growth and improve the sensitivity of immunotherapy in breast cancer.

## Data Availability

The data used to support the findings of this study are included within the article.

## Supplementary Materials

Supplementary Materials Supplementary Table 1: drug names from the Selleck drug library approved by the FDA.
