# Supplementary material for: Tegaserod Maleate Inhibits Breast Cancer Progression and Enhances the Sensitivity of Immunotherapy
Source: J Oncol. 2022 Feb 3;2022:5320421. doi: 10.1155/2022/5320421 (PMC8831063; doi:10.1155/2022/5320421)
Supplement: Supplementary Materials — Supplementary Table 1: drug names from the Selleck drug library approved by the FDA. [file 5320421.f1.docx]

Supplementary Table 1 Drug names from selleck drug library approved by FDA

| Chlorhexidine | Chlorhexidine 2HCl | Atovaquone | Pyridoxine HCl | Biotin (Vitamin B7) | Entacapone | Tylosin tartrate | Brinzolamide | Ropinirole HCl | Iopromide |
| --- | --- | --- | --- | --- | --- | --- | --- | --- | --- |
| Clofarabine | Piracetam | Etravirine (TMC125) | Vitamin C | Sulfamerazine | Estradiol valerate | Benztropine mesylate | Carbenicillin disodium | Ticarcillin sodium | Fexofenadine HCl |
| Dacarbazine | Vanillin | Ulipristal | Sulfathiazole | Sulfamethazine | Articaine HCl | Altrenogest | Eletriptan HBr | Azlocillin sodium salt | Moclobemide (Ro 111163) |
| Dexrazoxane HCl (ICRF-187, ADR-529) | Chlorthalidone | Indacaterol Maleate | Oxybutynin chloride | Sodium salicylate | Gliquidone | Ampicillin sodium | Flumequine | Reboxetine mesylate | Triptolide (PG490) |
| Epinephrine HCl | Dexmedetomidine | 2-Thiouracil | Doxepin HCl | Methylthiouracil | Butenafine HCl | Anagrelide HCl | Amitriptyline HCl | Triflusal | Borneol |
| Diclofenac Potassium | empty | Moguisteine | Ornidazole | empty | Mepivacaine HCl | Antipyrine | Adrenalone HCl | Trifluoperazine 2HCl | Fangchinoline |
| Diclofenac Diethylamine | Tazobactam | Nadifloxacin | Dexamethasone Acetate | Milnacipran HCl | Ethynodiol diacetate | Atomoxetine HCl | Azatadine dimaleate | Catharanthine | Berbamine (dihydrochloride) |
| Naloxone HCl | Beclomethasone dipropionate | Pidotimod | Trimethoprim | Darifenacin HBr | Sertaconazole nitrate | Betahistine 2HCl | (+,-)-Octopamine HCl | Meptazinol HCl | (+)-Fangchinoline |
| Levothyroxine sodium | Cinnamaldehyde | Echinacoside | Imperatorin | Panaxatriol | Harmine | Isopsoralen | Hederacoside C | Nonivamide | (+)-Borneol |
| Sodium benzoate | Tanshinone IIA sulfonate (sodium) | Notoginsenoside R1 | Scutellarin | D-Galactose | Guaiacol | Bornyl acetate | Lathyrol | Valproic acid | Vanillyl Butyl Ether |
| Quinidine sulfate | Palmatine | Carvacrol | Ginsenoside Re | Glucosamine sulfate | Indigo | Sophoridine | Ginsenoside Rg1 | L-Cycloserine | Nifuratel |
| 4-Hydroxybenzoic acid | 5-Hydroxymethylfurfural | Succinic acid | Harmine hydrochloride | Camphor | Scopoletin | Hydroxy Camptothecine | Ginsenoside Rb1 | Mesterolone | Flavone |
| Betaine | Tyrosol | Palmitic acid | Quercitrin | Tetrahydropalmatine hydrochloride | Protopine | Hederagenin | (-)-Epicatechin gallate | empty | Histamine |
| Methyl salicylate | Ligustrazine hydrochloride | Trigonelline Hydrochloride | Loganin | Allantoin | Pyrogallol | Astragaloside IV | Forsythin | Maltitol | Veratric acid |
| Sinomenine hydrochloride | cis-Anethole | Stevioside | Isoquercitrin | Lawsone | L-Rhamnose monohydrate | Catalpol | Swertiamarin | Tannic acid | Vindoline |
| Eucalyptol | Ginkgolide C | Dehydroandrographolide | Madecassoside | Galanthamine | Arteether | α-Hederin | Liquiritin | Gamma-Oryzanol | Fusidine |
| Parecoxib | Rebeprazole sodium | Gluconolactone | Rivastigmine | Vitamin K1 | Evans Blue | Perphenazine | Sulfacetamide sodium salt hydrate | Harmaline | Daminozide |
| Eslicarbazepine Acetate | Sivelestat sodium tetrahydrate | Povidone iodine | Deoxycholic acid | Etretinate | Isatin | Retigabine | Cisapride hydrate | Menadiol Diacetate | Thymidine |
| Hydroquinidine | Lidocaine hydrochloride | Terazosin HCl | Escin | 2-Deoxy-D-glucose | Acetylcholine iodide | Retigabine 2HCl | Corticosterone | Benzyl isothiocyanate | Ceftizoxime |
| Glycopyrrolate | Procaine | Protirelin | Oxybenzone | Eugenol | (+)-Catechin | Salvianolic acid B | Betulin | N-Acetylneuraminic acid | Cefuroxime axetil |
| Tiagabine hydrochloride | Benzocaine hydrochloride | Loxoprofen | Guanfacine Hydrochloride | Oleic Acid | (-)Epicatechin | Trapidil | Dihydrotestosterone(DHT) | Drostanolone Propionate | L-Cysteine HCl |
| Atazanavir | Etonogestrel | Sildenafil Mesylate | D panthenol | Latanoprost | Benzenesulfonamide | Psoralen | p-Coumaric Acid | Trenbolone acetate | Diatrizoate sodium |
| Fusidate Sodium | Hydroxyprogesterone caproate | Efavirenz | CarbinoxaMine Maleate | Esculetin | Lauric Acid | Ondansetron Hydrochloride Dihydrate | Melibiose | Methandrostenolone | Atenolol |
| Molsidomine | Tiagabine | Vitamin E | Saxagliptin hydrate | (-)-Menthol | Cinnarizine | Citalopram HBr | L-5-Hydroxytryptophan | Nicergoline | Saccharin |
| Diastase | Ibudilast | Faropenem Sodium | Iproniazid | Sulfamethoxazole sodium | Cefathiamidine | Cytosine | Maltol | Iminostilbene | Tiamulin fumarate |
| Maltose | Acotiamide hydrochloride | Dalbavancin | TriacetonaMine | Cefodizime Sodium | Calcium Dobesilate | Elagolix Sodium | Nonanoic acid | Fimasartan | Valpromide |
| Piperonyl butoxide | Mosapride | Levocetirizine Dihydrochloride | Indole-3-carboxylic acid | Pyridoxal 5-phosphate monohydrate | Lynestrenol | Sulfogaiacol | Fumaric acid | Sulfalene(SMPZ) | Methylcobalamin |
| Tolmetin | Laurocapram | Flucloxacillin sodium | Squalene | Cefazedone | Taurolidine | Propiverine hydrochloride | Usnic acid | Efonidipine | Tavaborole (AN-2690) |
| Cefoxitin sodium | Potassium acetate | Tafluprost | Cefetamet pivoxil hydrochloride | Cephapirin Benzathine | Menbutone | Proxyphylline | Linalool | Azathramycin | Avermectin B1(Abamectin) |
| Propantheline bromide | Cefcapene Pivoxil Hydrochloride | Gadopentetate Dimeglumine | Nicarbazin | Robenidine Hydrochoride | Nikethamide | empty | Glycocholic acid | Anamorelin | Tofacitinib (CP-690550) Citrate |
| Aceclofenac | Rabeprazole | Ecabet sodium | Propacetamol hydrochloride | Eperisone hydrochloride | Perospirone hydrochloride | Asunaprevir | Lactobionic acid | Sorbic acid | Fingolimod (FTY720) HCl |
| Nilutamide | Meropenem Trihydrate | Bedaquiline fumarate | Xanthinol Nicotinate | Neticonazole Hydrochloride | Bifendate | cis-Aconitic acid | Buparvaquone | empty | Tacrolimus (FK506) |
| Iguratimod | Diphenylpyraline hydrochloride | Drofenine Hydrochloride | Methyl linolenate | Phthalylsulfathiazole | Pravastatin | Oxantel Pamoate | Stearic acid | Deferoxamine mesylate | Citric acid |
| Hydroxyzine pamoate | Ertapenem sodium | Moxisylyte hydrochloride | Isoproterenol sulfate dihydrate | Alvimopan dihydrate | lurasidone | Dichlorophene | Midodrine hydrochloride | Morantel tartrate | Methyl Oleate |
| empty | Omeprazole Sodium | Fruquintinib | Desoximetasone | Fenipentol | atorvastatin | Triiodothyronine | Midodrine | Chlorpromazine | D-Mannose |
| Dexrazoxane | Emedastine | Isoxsuprine hydrochloride | Tropic acid | Carvedilol Phosphate | Abemaciclib | Tetryzoline | Benzathine penicilline | Dihydroergotamine Mesylate | Sodium Dehydrocholate |
| Elbasvir | Tiaprofenic acid | Chloropyramine hydrochloride | Mexenone | Raceanisodamine | Acetohexamide | Delapril Hydrochloride | Diclofenac Epolamine | Baricitinib phosphate | Alfuzosin |
| Indigo carmine | Ranitidine | Mivacurium chloride | Levomilnacipran Hydrochloride | Norgestrel | Acrivastine | Fosfosal | Nebivolol | Methyl Stearate | Aliskiren |
| Indacaterol | Minaprine dihydrochloride | Dolasetron Mesylate | Isopropamide Iodide | Ambroxol | Ceftizoxime sodium | Alimemazine Tartrate | Palonosetron | Isoeugenol | Fenoterol hydrobromide |
| Venlafaxine | Orphenadrine Hydrochloride | (-)-Verbenone | Ketorolac tromethamine salt | empty | Glecaprevir | Sebacic acid | Quetiapine | Methyl linoleate | Fenoterol |
| Nitisinone | Nerolidol | Butoconazole | Ethoxyquin | Betrixaban maleate | Dasabuvir(ABT-333) | Sultamicillin | Carbaryl | Hyperoside | Dantrolene sodium |
| Dolasetron | Cefpodoxime proxetil | Diflorasone | Ajmaline | Mepivacaine | Ombitasvir (ABT-267) | Ertugliflozin | Promazine hydrochloride | Saikosaponin D | Cloperastine hydrochloride |
| Meisoindigo | Cefmetazole sodium | Bendazac | Methyl Aminolevulinate Hydrochloride | Cyclofenil | Paritaprevir (ABT-450) | Diflucortolone valerate | Metoprolol | Curculigoside | Clidinium Bromide |
| Gamithromycin | Cefminox Sodium | Pikamilone | Dibutyl phthalate | Phenolphthalein | Propylparaben | empty | Quinacrine Dihydrochloride Dihydrate | Aucubin | Molindone hydrochloride |
| Ceftezole sodium | Cefpiramide sodium | Alogliptin | Dimethyl phthalate | Chlorhexidine | Sultamicillin Tosylate | Metyrapone | Berberine Sulfate | Saikosaponin A | Prilocaine hydrochloride |
| Sulbenicillin Sodium | Ceftiofur | Fipronil | Formate | Nefazodone hydrochloride | Squalane | Parecoxib Sodium | Triprolidine Hydrochloride | Pivmecillinam hydrochloride | Tribenzagan Hydrochloride |
| Metroprolol succinate | Safinamide | Ethyl Oleate | Imidafenacin | Chlorprothixene hydrochloride | Isoprene | 1,4-Cineole | Sofalcone | Rolapitant | Rimantadine Hydrochloride |
| Vanillic acid | Regadenoson | Lactitol | Betrixaban | Tegaserod Maleate | Chloramphenicol sodium succinate | Clindamycin alcoholate | Sanguinarine chloride | Gefarnate | Desipramine Hydrochloride |
| Cobimetinib (GDC-0973, RG7420) | Riociguat (BAY 63-2521) | Melphalan | Tirofiban Hydrochloride | Sanguinarine | Aristolochic acid A | Topotecan | Oxalic acid | Yangonin | empty |
| Venetoclax (ABT-199, GDC-0199) | Sivelestat (ONO-5046) | Olmutinib (HM61713, BI 1482694) | Sodium dichloroacetate (DCA) | Wedelolactone | Melamine | Scopolamine | ADP | Uridine 5'-monophosphate | Demecarium Bromide |
| Macitentan | Halofuginone | Erdafitinib (JNJ-42756493) | Ipragliflozin (ASP1941) | Berberine | Dipotassium glycyrrhizinate | Carboprost | Neryl acetate | Undecanoic acid | Methysergide Maleate |
| Vorapaxar | Mitomycin C | Troglitazone (CS-045) | Anlotinib (AL3818) dihydrochloride | Harringtonine | Sinensetin | Orcinol | 5,7-Dihydroxy-4-methylcoumarin | Phensuximide | Methenamine Hippurate |
| CB-5083 | Pimavanserin | TAS-102 | Malic acid | Pulegone | Isofraxidin | Octyl gallate | Thymine | Ergoloid Mesylates | Thiothixene |
| Acalabrutinib (ACP-196) | Oclacitinib?maleate | Tofogliflozin(CSG 452) | L-Fucose | Berbamine | Securinine | 1-Indanone | Methyl palmitate | Mecamylamine Hydrochloride | Haloperidol Decanoate |
| Resiquimod | Enasidenib (AG-221) | Omarigliptin (MK-3102) | (R)-(-)-Mandelic acid | Sparteine | 3-n-Butylphathlide | D-(+)-Raffinose pentahydrate | Ligustilide | Ethotoin | Penbutolol Sulfate |
| Radotinib | Ivosidenib (AG-120) | Tucidinostat (Chidamide) | 2'-Deoxyguanosine monohydrate | Ammonium Glycyrrhizate | Germacrone | 2'-Deoxyadenosine monohydrate | Rhynchophylline | Benzonatate | Oxtriphylline |
| Axitinib | Gefitinib (ZD1839) | Sorafenib Tosylate | Crizotinib (PF-02341066) | Docetaxel | Anastrozole | Cladribine | Methotrexate | Letrozole | Entecavir Hydrate |
| Roxadustat (FG-4592) | Imatinib Mesylate (STI571) | Sunitinib Malate | Vismodegib (GDC-0449) | Paclitaxel | Aprepitant | Decitabine | Bendamustine HCl | Temozolomide | Nepafenac |
| Nintedanib (BIBF 1120) | Lapatinib (GW-572016) Ditosylate | Temsirolimus (CCI-779, NSC 683864) | Belinostat (PXD101) | Capecitabine | Bicalutamide | Dutasteride | Epirubicin HCl | Tamoxifen | Rufinamide |
| Afatinib (BIBW2992) | Lenalidomide (CC-5013) | Vorinostat (SAHA, MK0683) | Rucaparib (AG-014699,PF-01367338) phosphate | Lenvatinib (E7080) | Fulvestrant | Melatonin | Etoposide | Vincristine sulfate | Posaconazole |
| Bortezomib (PS-341) | Panobinostat (LBH589) | Entinostat (MS-275) | Cabozantinib (XL184, BMS-907351) | Valproic acid sodium salt (Sodium valproate) | Raltitrexed | Bisoprolol fumarate | Raloxifene HCl | Agomelatine | Prasugrel |
| Bosutinib (SKI-606) | Nilotinib (AMN-107) | Enzastaurin (LY317615) | Everolimus (RAD001) | Regorafenib (BAY 73-4506) | Thalidomide | Tivozanib (AV-951) | Fludarabine Phosphate | Leflunomide | Ramelteon |
| Dasatinib | Pazopanib HCl (GW786034 HCl) | Olaparib (AZD2281, Ku-0059436) | Malotilate | Danoprevir (ITMN-191) | Exemestane | Doxorubicin (Adriamycin) HCl | Topotecan HCl | Enzalutamide (MDV3100) | Cinacalcet HCl |
| Ridaforolimus (Deforolimus, MK-8669) | Rapamycin (Sirolimus) | Masitinib (AB1010) | Ivacaftor (VX-770) | Ritonavir | Finasteride | Fluorouracil (5-Fluoracil, 5-FU) | 2-Methoxyestradiol (2-MeOE2) | Dienogest | Celecoxib |
| Ketorolac | Enalaprilat Dihydrate | Aminoglutethimide | Ipratropium Bromide | Hydrocortisone | Deferasirox | Azathioprine | Meloxicam | Nevirapine | Pitavastatin Calcium |
| Adenosine | Dofetilide | Aminophylline | Sulfanilamide | Desonide | Piroxicam | Indomethacin | Mesna | NEXIUM (esomeprazole magnesium) | Rifapentine |
| Zolmitriptan | Isradipine | Lubiprostone | Betamethasone Dipropionate | Didanosine | Gemcitabine | Terbinafine | Methocarbamol | Nicotinic Acid | Suprofen |
| Telbivudine | Estrone | Amorolfine HCl | Meprednisone | Divalproex Sodium | Glipizide | Levonorgestrel | Prednisolone | Nimodipine | Pyrazinamide |
| Monobenzone | Flucytosine | Chloramphenicol | Betamethasone Valerate | Emtricitabine | Glyburide (Glibenclamide) | Gemfibrozil | Telmisartan | Nisoldipine | Quetiapine Fumarate |
| Tretinoin | Trichlormethiazide | Flurbiprofen | Praziquantel | Progesterone | Fomepizole | Indapamide | Thiabendazole | Octocrylene | Rifampin |
| Phenylbutazone | Loteprednol etabonate | Disulfiram | Busulfan | Lamivudine | Adefovir Dipivoxil | Mitotane | Guaifenesin | Oxybutynin | Cefditoren Pivoxil |
| Ezetimibe | empty | Mesalamine | Carbamazepine | Estradiol | Zalcitabine | Methylprednisolone | Rifabutin | Enoxacin | Sulfadiazine |
| Clindamycin palmitate HCl | Buflomedil HCl | Clinofibrate | Canagliflozin | Dabrafenib (GSK2118436) | Alogliptin（SYK-322）benzoate | Icotinib | Amoxicillin | Fenoprofen calcium hydrate | Cinepazide maleate |
| L-Thyroxine | Fluocinonide | Ciprofibrate | Alectinib (CH5424802) | MPEP | Camostat Mesilate | Carbazochrome sodium sulfonate (AC-17) | Aspirin | Linagliptin | Otilonium Bromide |
| Gliclazide | Inulin | Dolutegravir (GSK1349572) | MK-2048 | Alpelisib (BYL719) | Prucalopride | Clevudine | Niflumic acid | Vildagliptin (LAF-237) | Bosentan Hydrate |
| Acemetacin | Lonidamine | Trametinib (GSK1120212) | Laquinimod | Clindamycin | Acesulfame Potassium | Rivaroxaban | Ciclopirox ethanolamine | Daunorubicin HCl | Rupatadine Fumarate |
| Tioxolone | Clorsulon | Ibrutinib (PCI-32765) | Tofacitinib (CP-690550,Tasocitinib) | Epiandrosterone | Cobicistat (GS-9350) | Prostaglandin E2 (PGE2) | Rimonabant | Pravastatin sodium | Azelnidipine |
| Dehydroepiandrosterone (DHEA) | Arecoline HBr | Nilvadipine | Istradefylline | Apalutamide?(ARN-509) | S-Ruxolitinib (INCB018424) | Paroxetine HCl | Cabazitaxel | Bepotastine Besilate | Alverine Citrate |
| Idebenone | Noradrenaline bitartrate monohydrate | Dacomitinib (PF299804, PF299) | Torcetrapib | Baricitinib (LY3009104, INCB028050) | Lumiracoxib | Zaltoprofen | Bufexamac | Fosaprepitant dimeglumine salt | Azilsartan Medoxomil |
| Mifepristone | Fostamatinib (R788) | Niraparib (MK-4827) | Sofosbuvir (PSI-7977, GS-7977) | Carfilzomib (PR-171) | Pirfenidone | Pazopanib | Lamotrigine | Rofecoxib | Medetomidine HCl |

**Empty means no drug in this hole.**
